# HipHop2SToP a community-led health promotion initiative empowering Aboriginal youth in the Kimberley region of Western Australia: a process evaluation

**DOI:** 10.3389/fpubh.2023.1258517

**Published:** 2023-12-08

**Authors:** Tracy McRae, Roz Walker, Stephanie Enkel, Hannah M. M. Thomas, John Jacky, Slade Sibosado, Marianne Mullane, Natasha Maginnis, Juli Coffin, Jonathan R. Carapetis, Asha C. Bowen

**Affiliations:** ^1^School of Medicine, University of Western Australia, Perth, WA, Australia; ^2^Wesfarmers Centre of Vaccines and Infectious Diseases, Telethon Kids Institute, Nedlands, WA, Australia; ^3^Ngangk Yira Institute For Change, Murdoch University, Perth, WA, Australia; ^4^School of Indigenous Studies, Poche Centre for Indigenous Health Research, University of Western Australia, Perth, WA, Australia; ^5^School of Population and Global Health, University of Western Australia, Perth, WA, Australia; ^6^Telethon Kids Institute, Nedlands, WA, Australia; ^7^Department of Infectious Diseases, Perth Children’s Hospital, Nedlands, WA, Australia; ^8^Menzies School of Health Research, Charles Darwin University, Darwin, NT, Australia; ^9^University of Notre Dame, Fremantle, WA, Australia

**Keywords:** health promotion, Aboriginal health, skin infections, hip-hop music, community-led

## Abstract

**Introduction:**

For millennia, Aboriginal people’s ways of knowing, doing and being were shared through art, song, and dance. Colonisation silenced these ways, affecting loss of self-determination for Aboriginal people. Over the past decade in Australia, hip-hop projects have become culturally appropriate approaches for health promotion. When community led, and Aboriginal worldviews centralised, hip-hop workshops are more likely to be effective. In 2020, during the COVID-19 pandemic, a community-led health promotion hip-hop music video, ‘HipHop2SToP’ was produced involving young people in Dampier Peninsula communities address healthy skin and healthy living practices.

**Methods:**

We report here a qualitative process evaluation of the HipHop2SToP project. Participants who had been involved in the planning and production of HipHop2SToP were selected using a purposive approach and invited either by email or face-to-face to participate in semi-structured interviews and share their experiences. Semi-structured interviews ranged from 30 to 60 min in duration and were conducted either face-to-face or virtually over MS Teams. Due to personal time constraints, two participants provided written responses to the semi-structured questions. All interviews were audio-recorded with consent and saved as a digital recording in a de-identified format. All audio recordings were transcribed verbatim and uploaded into QSR NVivo v12 along with written responses.

**Results:**

As a health promotion project, the critical success factors were community-ownership and discovering novel ways to collaborate virtually with remote communities using Microsoft (MS) software. Highlights included observing the young people actively engaged in the project and their catchy lyrics and key messaging for environmental health and skin infections. COVID-19 presented some challenges. Gaps in communication, clarification of stakeholder roles and expectations, and post-production outcomes were also identified as challenges.

**Conclusion:**

HipHop2SToP validates the need for Aboriginal community led health promotion programs. While creating some challenges COVID-19 also strengthened community ownership and created novel ways of maintaining relationships with remote Aboriginal communities. Future hip-hop projects would benefit from clarity of roles and responsibilities. Strengthening post-production outcomes by including a launch and well-planned, targeted communication and dissemination strategy will ensure the wider translation of important health messages and potential strengthen sustainability.

## Introduction

For Millenia, Australian Aboriginal people’s ways of knowing, doing and being have been shared through art, song, and dance (1). This oral tradition of story sharing, and expression of voice encompassed a wide range of knowledges, ensuring generations inherited a broad and interconnected understanding of their culture and environment (1, 2). Through the process and effects of colonisation, these ways of knowing, being and doing were silenced, affecting the loss of self-determination for Aboriginal people (3). In doing so, Euro-colonial epistemologies dominated institutions, systems, and research, perpetuating ongoing power imbalances and systemic racism (4, 5); thus health promotion has been based on Euro-colonial epistemologies and considered a colonial language denying the worldviews of Aboriginal people (6). In doing so, an authoritative dominance and control over Aboriginal people’s lives has been fostered by way of instructing Aboriginal people on what they should and should not do for their own health, rather than give options to ‘choose’, inform and co-design the best messages for themselves (7, 8). Furthermore, these power dynamics and paradigms have marginalised already vulnerable populations (9, 10). Transcending post-colonial health promotion into strength-based programs among Aboriginal communities requires a paradigm shift that centralises Aboriginal worldviews. This transformation is essential for Aboriginal peoples’ self-determination (6, 11) and reducing the burden of disease affecting their families and communities (12).

### Reducing the burden of skin infections

The burden of skin disease remains high in Aboriginal populations, particularly, Aboriginal children who experience higher rates of skin infections compared to non-Aboriginal children (13). The two main skin infections of concern are scabies, an itchy skin infection transmitted between humans (14) via skin-to-skin contact and impetigo which often develops as a secondary bacterial infection from scabies or other broken skin or following minor trauma (15). In 2017 the World Health Organization (WHO) included scabies in the program of Neglected Tropical Diseases (16, 17), establishing a global drive for the promotion and implementation of scabies control programs in endemic countries. Acknowledging the broader social and environmental determinants of health and local conditions in which people are born and live is crucial for reducing the burden of skin disease (18). Engaging with, listening to, and accepting guidance from community Elders to learn their wisdom, traditions, and unique Aboriginal worldviews is essential for effective programs (9). For non-Aboriginal organisations, adopting a critical lens to interweave the rich Aboriginal knowledge systems with biomedical knowledge and technology (19), is a positive approach for health messages to be delivered in culturally sensitive and relevant way. When done effectively, this approach can help increase participant engagement, facilitate active learning, raise awareness (20–22), and increase confidence and social inclusion for those involved (20–23). Over the last decade in Australia, this has been demonstrated in workshops and programs using hip-hop music as a means to engage Aboriginal young people (20, 21, 23–25). Creating hip-hop music and participating in workshops has provided healthy ways for Aboriginal young people to make meaning of themselves and their life experiences (23), and facilitate emotional regulation (26). Furthermore, hip-hop music has demonstrated its power for representing identity (27), and self-expression, in addition to providing therapeutic functions (23, 28) and maintaining traditional ways of knowing, doing and being through song and dance (24).

#### HipHop2SToP – community led health promotion initiative within the see treat prevent trial

Acknowledging the importance of Aboriginal community led programs, the purpose of this paper is to report on a community led health promotion hip-hop music initiative, namely HipHop2SToP implemented in four Dampier Peninsula communities. Under guidance from Woombooriny Amboon Angarriiya Partnership Initiative (WAAPI) Community Navigators^1^ HipHop2SToP is an example of a community led, strengths-based approach to health promotion situated within a broader research project, See, Treat, Prevent (SToP) Trial: a randomised clinical trial aimed at reducing skin infections by 50% in children living in the Kimberley, Western Australia (29). In May 2019, SToP Trial activities commenced including face-to-face school-based skin surveillance; clinic treatments streamlined to include the latest evidence-based practice; and consultation with participating communities to co-design individualised health promotion initiatives. Well-recognised and popular music amongst the young people living in the Dampier Peninsula, a hip-hop music video was chosen by WAAPI community navigators, and supported by Elders as a culturally appropriate, and meaningful SToP Trial health promotion initiative to raise awareness on environmental health and skin infections. Community led and locally produced, HipHop2SToP was planned during virtual meetings and then produced face-to-face during the COVID-19 pandemic.

### Hip-Hop subculture, music, and projects

Originating in the United States of America (USA), hip-hop music evolved as a self-expressive artform for many marginalised populations including African American youths (30–32). During the 1980s and 1990s, African American youths relished hip-hop music as an enlightened way of making sense of their world and the reality of life in their neighbourhoods and urban ghettos (30). The hip-hop subculture transcended into popular music industries, becoming a global language (32), and an example of how Indigenous populations globally utilised their cultural traditions of art, music, and dance as modern forms of storytelling. External organisations such as Indigenous Hip-Hop Projects (IHHP) have engaged with Aboriginal communities and young people across Australia, delivering workshops and producing music videos covering topics that include but are not limited to skin health (33), sexual (20) and mental health (21), and crime (34). These projects have facilitated a space for young people to actively engage and learn. For remote living Aboriginal young people involved in music workshops focused on sexual and mental health, hip-hop music helped to increase their social awareness and self-confidence, strengthen communities, and promote social change (20, 21). Further, the young people involved in the *beyondblue: the national depression initiative*, believed hip-hop music provided a means for reducing shame around mental health and increased their confidence to speak with peers and family regarding depression and anxiety (21). Similarly, high rates of participation in the sexual health focused hip-hop project was shown to increase young people’s confidence and encourage them ‘out of their shell’ (20).

Desert Pea Media (DPM), a cross-cultural organisation, has worked with over 80 remote and regional communities in Australia delivering workshops and engaging with young people (25). Between 2019 and 2021, DPM delivered a series of mental health focused workshops in New South Wales and Queensland. These workshops created a safe space enabling young people to ‘step up’ and try something new. Participating in these workshops strengthened identity and increased the young people’s sense of worth. Further, these workshops enabled open discussions about mental health among participants, breaking down the stigma often associated with this topic (22). Furthermore, studies in the USA report emotional and well-being benefits for participants involved in hip-hop summer camps (26, 28). These included social growth and awareness where the participants collaborated with their peers, experiencing joy in creating meaningful projects together.

Despite positive aspects and influence for participants, hip-hop workshops and projects have not been without criticism in terms of further exploitation and paternalism (35). Genuine collaborations in all elements of hip-hop workshops can help mitigate this, and reduce the risk of power imbalances and colonial relationships when non-Aboriginal organisations work in this context (24). Hip-hop projects can help raise awareness on the subject of interest, however, concerns regarding sustainability and longer-term benefits have been reported on projects with limited community engagement and prior planning (20, 21). Furthermore, staff involved in hip-hop projects have reported issues regarding lack of clarity on roles and responsibilities (20). Well-designed projects with established aims and goals, and consideration for appropriate facilities and project timeframes are more likely to be effective (23). Aboriginal engagement and leadership throughout programs are key elements, as is respecting self-determination (11, 23, 24). Without these components, programs are less likely to be successful in translating health messages or influencing health outcomes.

### COVID-19 and community leadership

COVID-19 is an important factor in HipHop2SToP, highlighting community strength and leadership in all elements of the project and the novel way the pandemic connected people using digital technology such as Microsoft (MS) Teams and Zoom when international and interstate travel restrictions were enforced by Federal and State governments (36). At this time, and following direction from Aboriginal leaders, remote Aboriginal communities were closed to all visitors (37). For the SToP Trial, COVID-19 travel restrictions meant all face-to-face school-based skin surveillance and engagement with communities were no longer an option, raising valid concerns that SToP Trial activities would cease indefinitely. This also created concerns around maintaining relationships with communities given the inability to meet face-to-face. Fortunately, online meetings using digital technology became normalised, and communities were receptive to yarning^2^ (38) virtually via MS Teams.

Connecting virtually with the WAAPI community navigators and the process of planning the camp and music project has been reported in-depth elsewhere (39) however, bringing the young people together virtually to write the lyrics presented initial challenges due to the communities remaining closed. Fortunately, with support from the schools, eight virtual song-writing workshops prior to the camp were organised. These after-school virtual workshops were conducted in two Dampier Peninsula schools with SToP Trial team members in Perth, a local hip-hop artist in Broome and two hip-hop artists from Melbourne joining in via Zoom.

### Lyrics for environmental health and skin infections

Aligning with the theme of skin health and environmental health, the young people learnt about the nine Healthy Living Practices (HLPs) (40) that guided key messages in the lyrics. Outlined in Table 1, these guidelines define the essential living conditions required to ensure people are able to live healthy lives. Conception of the HLPs resulted from a request by Nganampa Health Council directors who provided health services to Aboriginal people in the Aṉangu Pitjantjatjara Yankunytjatjara (APY) Lands to ‘*stop people getting sick*’. The HLPs highlight the need for both ‘health hardware’ (41, 42) (e.g., functioning showers and taps, availability of a washing machine) and ‘health software’ (e.g., towels, soap, mattresses) within the home, while highlighting personal strategies one can employ to keep themselves free of infectious diseases. Although concepts from HLPs were embedded into the lyrics of the song, the young people created lyrics that were meaningful to them, including words such as Gubinge (Kakadu Plum) in the chorus. The young people also initiated a narrative, not related to skin health but about keeping heart, spirit, and connection strong and sung the words - ‘*Keep your ‘Liyarn’ strong, Hope you enjoy our song*’ at the end of the song.

**Table 1:** Healthy living practices.

HLP 1	Washing people
HLP 2	Washing clothes and bedding
HLP 3	Removing wastewater safely
HLP 4	Improving nutrition, the ability to store prepare and cook food
HLP 5	Reducing the negative impacts of over-crowding
HLP 6	Reducing the negative effects of animals, insects, and vermin
HLP 7	Reducing the health impacts of dust
HLP 8	Controlling the temperature of the living environment
HLP 9	Reducing hazards that cause trauma

While the pandemic required innovative ways to plan the camp and produce HipHop2SToP, it also strengthened the community-driven approach and cultural integrity of the project through employing local people and contracting local organisations rather than externally outsourcing personnel. Over 20 community members (3 of whom were employed by the SToP Trial) supported the camp and provided supervision, 15 service providers and stakeholders facilitated workshops, and 41 young people attended the camp. While remote communities remained closed to non-essential visitors, travel within WA was permitted therefore a neutral location in the Dampier Peninsula that allowed face-to-face engagement was selected for the camp. With restrictions lifting to travel within WA, four staff from the SToP Trial and Kulunga^3^ teams who were involved in planning, were able to attend the five-day camp in person. There has been an influx of hip-hop projects in Australia in the past decade, of which several have been evaluated and contribute to the existing literature (20–24). We aim to add to the existing literature on hip-hop projects, highlighting the strengths of community leadership during COVID-19. Furthermore, there is limited research reporting locally produced hip-hop music videos in the Dampier Peninsula region of WA. Consequently, the current research project reports the perspectives of SToP Trial and Kulunga staff, community navigators and local organisation staff who were involved in the planning and implementation of the camp and HipHop2SToP music video. To achieve this aim, this project has two objectives:

1. To understand how project facilitators and challenges impacted planning and implementation of the music video.
2. To identify highlights and recommendations for future community led projects.

## Methods

Underpinned by the philosophy of constructivism (43), we report here a qualitative process evaluation (44) of the HipHop2SToP community led health promotion initiative. Participants were selected using a purposive (45) approach and invited by TM either by email or face-to-face to participate in semi-structured interviews. These semi-structured interviews provided an opportunity for participants who had been involved in the planning and/or producing of HipHop2SToP to describe their experiences and share their perspectives on the strengths and challenges of the project. All participants were provided with information pertaining to the research project, assured of confidentiality, and advised that participation was voluntary prior to providing written informed consent. Semi-structured interviews ranged from 30 to 60 min in duration and were conducted by TM either face-to-face in Perth or Broome or virtually over MS Teams. Due to personal time constraints, two participants provided TM with written responses to the semi-structured questions.

All interviews were audio-recorded with consent and saved as a digital recording in a de-identified format. All audio recordings were transcribed verbatim and uploaded into QSR NVivo v12 (46) along with written responses. Each transcript was assigned a code number to protect participant privacy. Adhering to the question guide, the transcripts and written responses were coded independently following the broad topic areas and specific theme codes were added where new themes emerged from the data. Situated within the Patient, Provider and Practice (P3) framework (47), an inductive process was undertaken for themes emerging from the analysis. An appropriate framework to evaluate SToP Trial activities given the See, Treat, Prevent elements, the P3 framework facilitates triangulation of data from SToP Trial staff (practice and provider) and Kulunga staff (practice and provider), community navigators (patient, practice, and provider) and local organisation staff (patient, practice, and provider). The P3 framework not only conceptualises their personal worldviews (both Aboriginal and non-Aboriginal), but also considers the broader systems and policies. This is particularly important given the context of COVID-19, travel restrictions and community closures.

### Ethics

This project was approved by the health ethics review committees at the Child and Adolescent Health Service (Approval number RGS0000000584), the Western Australian Aboriginal Health Ethics Committee (Reference number: 819), University of Western Australia (Reference RA/4/20/4123), Catholic Education Western Australia (Reference number: RP2017/57) and Western Australian Department of Education (Reference number: D18/0281633).

## Results

Eight participants were interviewed in this research project (seven females and one male), with four participants identifying as Aboriginal. Respondents included community navigators, researchers, and employees of local organisations, who described their own experiences of the HipHop2SToP project and reflected on the highlights and challenges of this initiative. Major themes emerging from the data revealed critical success factors as community-ownership; youth empowerment; and discovering novel ways to collaborate virtually using Microsoft (MS) and Zoom software. In addition to COVID-19 and associated travel restrictions and community closures; gaps in communication, clarification of stakeholder roles and expectations, and post-production outcomes were identified as challenges. Several participants discussed the key messaging and sustainability aspects of HipHop2SToP, highlighting local children continued to sing certain lines of the song and make the connection between healthy living practices and skin infections. Advice for future strategies included the importance of having appropriate mechanisms for directly employing local community members.

## Strengths

### Community leadership

All participants believed the critical success factor of HipHop2SToP was the role of the Dampier Peninsula communities in leading the project, under the guidance of the WAAPI community navigators. All participants recognised the effective leadership and guidance that community navigators provided to all stakeholders involved. While a research health promotion initiative, several participants articulated their appreciation for the culturally appropriate and respectful way that the research team worked alongside WAAPI, strengthening the integrity of the community led initiative.

“I don't think that the outcomes would be the same and I don't think the promotional message would've got across as clearly … the community have a way of speaking to community and they have a way of gathering the community and here’s a clear example of the need for it to be community driven”. Local Organisation Staff

“I think the key thing was the community control in planning and giving that directive and just, you know, the coordination from the ground.” Community Navigator

“I thought Telethon Kids staff worked really well with [the] navigators of the communities … they were given a lot of creative licence to lead [the video] work around the kind of loose general script on the purpose of the video to raise awareness around prevention of skin sores and skin health but letting the kids kind of define lyrics that they felt would resonate most about what seemed to work really well and so the cultural integrity of the whole project was really solid because of that”. Local Organisation Staff

### Learning novel ways of working

While COVID-19 travel restrictions presented challenges for the planning and implementation of the camp and music video, COVID-19 also provided novel ways of working together despite the large geographical distance between stakeholders. From a research perspective, COVID-19 forced researchers to rely on and trust the community to facilitate HipHop2SToP, and this showcased the true power, resilience, and effectiveness of community led projects. This was reflected from a community navigator perspective, where COVID-19 had minimal effect as they continued to plan the project on the ground in their communities at a time when service providers and visitors were unable to visit. Responding to COVID-19 led to novel ways for all stakeholders to connect virtually via MS Teams software and enabled a community led approach to conducting research. This novel approach also enabled the young people to engage in virtual workshops after-school, putting their own ideas and cultural lens into the lyrics.

“I think there was very minimal effect from the COVID and that's purely because of the community navigators holding a core position within community and having the capacity and resources to still engage with stakeholders out of the community and still having that position within the community to still do the community engagement.” Community Navigator

“Look I feel like we've learnt a lot during COVID and thankfully we have the sort of technology even on the Peninsula you know like in these beautiful remote areas, we were still able to connect with the kids who, who directed the writing of all the lyrics” Local Organisation Staff

“All of a sudden with COVID times [meeting face-to-face was] no longer possible so a lot of the work for the HipHop2SToP video happened via MS teams meetings so I think that planning the song writing and bringing together people from Melbourne, and Broome and Perth as well as the communities together to write the song via MS teams was a uniquely COVID experience”. Telethon Staff member

## Challenges

### Communication and clarity of roles

The community led camp and music project was a large collaboration involving approximately 100 people including the young people attending the camp. While those involved were able to meet virtually to discuss planning, the challenges of being unable to meet face-to-face were identified. Telethon Kids Institute and local organisation staff described difficulty at times navigating all the conversations and having clarity on the roles and responsibilities of all stakeholders involved. Clarity about the leadership of the camp and the hip hop video (as a sub-activity of the overall camp) was challenging to establish and was re-negotiated frequently before, during and after the camp. Whilst this was a challenge, it was inevitable that this style of working together, across various organisations, geographical difference, age ranges and cultures would result in endless conversations to discover who needed to take responsibility for which aspects. Much of this also occurred during the week of the camp as emerging priorities led to daily changes. The wellbeing and needs of the young people remained central and enabled these conversations to facilitate productive resolutions and great outcomes for all.

“I think over the course of the hip-hop there was probably over 100 different people involved from the kids, the 35 or so kids who were at the camp to the community leaders, to the CNs to the different agencies, with that many people involved there was certainly going to be some gaps.” Telethon Staff member

“I s’pose the distance between the home base of the SToP team which is really in Perth and then in Broome was hard to sort of get everyone in the same room to meet and talk, discuss it really, really in depth and yeah [being kept] in the loop with everything on my end was tough because there were conversations being had where we couldn't necessarily always be involved in and being sort of on the front line with it, it was yeah, tough trying to manage all of those conversations.” Telethon Staff member

“[In future] we have to get it right. We have to make sure that we map out who are all the stakeholders from the outset and make sure that we're continuously checking as to who is being consulted and who's being informed.” Local Organisation Staff

“I felt it was a great effort in the little time we had to plan. It was challenging to know who was leading the project at times and who was responsible for what.” Local Organisation Staff

### No official launch

Despite the critical success factor of community led project facilitation, COVID-19 and other priorities presented challenges post-production. Whilst acknowledging the difficulties with COVID-19 restrictions, participants described their disappointment with the post-production outcome overall, particularly with not having an official launch to celebrate what had been achieved during a challenging time. Despite this, access to the video via YouTube has ensured frequent viewings by individuals, school classes, community groups and the wider public. The video has been viewed >4,000 times on YouTube and other social media platforms.

“There hasn't been the launch, and again post-production I feel like we had a great team, the sound guy did his job brilliantly which synced all of the video footage to the audio footage so post-production I think all worked well but there has not been an official launch”. Local Organisation Staff

“There was no one who took charge of finishing up the product and so I think that was a function of time, but I do think that we dropped the ball at that point in time and it was quite hard to manage the relationships as well as the ability to do a launch.” Telethon Staff member

“Yeah, again COVID-19 sort of restricted a lot of what [we] could and couldn't do in regard to accessing community and really trying to pump it up and pump up the ah the involvement with the kids you know, it would’ve been nice for us to have [an] official event to launch it you know and really make a spectacle out of it with the community but unfortunately restrictions were pretty hard and on and off in Perth as well.” Telethon Staff member

## Highlights

### Learning about environmental health and skin infections

HipHop2SToP was a health promotion initiative and when asked about the healthy skin messaging embedded within the lyrics, several participants reported their joy in seeing the lyrics still resonating with the local Dampier Peninsula children. Participants believed the production of the music video facilitated a space for the young people to actively engage and learn practical ways to keep skin healthy. Key messages created by the young people were developed during the lyric writing workshops and reinforced throughout the week of production by the young people ‘learning by doing’.

“Yep, well in Aboriginal communities everyone knows that song and dance is the main way of us learning and learning by doing so if you got your own people on the screen doing things and singing about it, I think the message sinks in you know there's different forms, people like doing like art, music is an art.” Telethon Staff member

“Um so it made me very, very happy, it made my heart happy last week when I was told that a young boy was walking from the shower or to the shower singing 'gotta have a shower, to look after my power' and singing the lyrics of the song and that is really, that's just what we want, that was the outcome we wanted.” Telethon Staff member

“oh yeah absolutely, the lyrics were really clear, they were wonderful lyrics, and the messages were really clear, and I think it worked really well because you know they [the children] had their own spin on it, it was on their country, it was their culture, they were promoting having fun with their local cool hip hop artist but still the messaging was clear, in their own language and cultural kind of lens for it so it worked really well.” Local Organisation Staff

“… [I was] sitting in the clinic waiting room [and a parent commented to me] whatever you do you gotta keep this camp going because [the children] came back so excited and they just can't stop talking about the music video and then they're actually singing it.” Community Navigator

### Empowerment and ownership

The success of having community leadership provided what the participants felt was a space for the young people involved in the camp and music project to be confident. While the interviewees had varying roles, when reflecting on the highlights of the camp and music project they all expressed their delight in observing the young people strengthening their confidence, exhibiting their creativity, engaging in all camp activities, and taking ownership of the music video.

“such a beautiful experience for myself and for the kids to see that, to have the experience of owning their own voices, I mean that's huge and also the confidence that is required to present yourself on the camera, are all confidence building skills” Local Organisation Staff

“I honestly, really loved the music workshops previous to the camp … the things that stood out to me as part of this whole process was the hip hop music video …, it was their idea [to meet] [Telethon staff and music artists] and it was like OK, we can do online stuff … that activity was really good to see the kids coming in, they were giving their own time straight after-school and just [the] expressions on their faces, you know that they were actually writing the song themselves and they were gonna sing it, helping produce it, so the idea, so that thing about empowering the youth is a big thing”. Community Navigator

“I really enjoyed the hip hop video itself, seeing those kids working in different groups and then when it all came together and they started to sing in the final days and everyone was standing around listening to them, see like them having a lot of fun and we could see it all being pulled together you know you could really sense everyone's excitement that [we] pulled this off and so much professionalism around with [organisation name] the artist and all working together”. Local Organisation Staff

“I think we all sort of got the same sort of highlight right towards the end you know when the kids really came out of their shell and were getting right into all of the activities especially the hip-hop”. Telethon Staff member

## Future strategies

### Organisational processes, contracts, and systems

When discussing what could be done differently or improved on for future projects, participants reflected on lessons learnt, focusing on the importance of ensuring formal agreements were developed to clarify roles and responsibilities at the outset of the project. This was a factor of both the camp and music video being new initiatives that came together with the additional challenges of travel restrictions during COVID-19. Despite the project evolving over many months of planning, all participants agreed that early attention to formal agreements would have strengthened the initiative. In addition, Telethon Kids Institute staff in particular discussed the need for having appropriate mechanisms for directly employing local community members. As the project was funded as a research activity, it was important to the researchers to employ local community members to lead the music video. However, this was challenging with the overlap of the camp led by WAAPI, and with most of the community members employed by this mechanism. Demonstrating reciprocity and respectful engagement in research is a high priority – and local employment is one strategy raised by local community members frequently to achieve this. This is why it was such a priority for the research staff to find ways to achieve this.

“I felt conflicted throughout the process and believe a clear agreement should have been negotiated and confirmed in writing rather than verbally.” Local Organisation Staff

“I think the first and foremost one is really having at the [organisation name] a clear way in which we can appropriately employ research assistants as community navigators in community and I think that's been the goal of many of our discussions this year.” Telethon Staff member

“[How to employ] the navigators directly because [they were] engaged through [organisation name] who we engaged with and for, on all accounts they we're employed as a [local organisation] employee so it was hard to know the line for them on where they fell under a Telethon Kids employee or whether they were still under a [local organisation] employee.” Telethon Staff member

“I think we all learnt from this process about the importance of having clear communication about what we're doing as the activity but also about who is responsible and accountable.” Telethon Staff member

## Discussion

Our study provides unique insights into the facilitators and challenges experienced during the planning and production of the WAAPI community led HipHop2SToP music project during the COVID-19 pandemic. The critical success factor and major highlight for HipHop2SToP was the ground-up, community led approach that empowered local youth and communities. A community led approach to help reduce the burden of disease in Aboriginal communities has been well documented (12) and validated in our study. A strong sense of Aboriginal knowing, doing and being underpinned the philosophy of the youth empowerment camp and music video, strengthening the cultural validity and integrity of the project.

HipHop2SToP is an example of using the art of song and dance to move beyond Euro-colonial epistemologies and create inclusive approaches for where Aboriginal worldviews are centralised (22–24). Similar to the experience of the young people involved in a previous sexual health project (20), the HipHop2SToP environment encouraged the youths ‘out of their shells’ to embrace their creativity. Interviewees indicated this art form provided a source of strength and engagement for health promotion messages, disseminated through a cultural and creative lens that has been used and evaluated for their contribution to health promotion (20, 21). Underpinned by environmental health and healthy skin science, the young people took ownership of the key messages, developing catchy lyrics that continue to resonate with the young people and communities of the Dampier Peninsula. Our findings move beyond the limited community engagement reported in some projects (20) and validate the need for community led health promotion where Aboriginal people’s voices and culture are central to within the messaging to help facilitate ownership and strengthen sustainability (23, 24).

COVID-19 also highlighted and encouraged opportunities for local people to be employed and local organisations to be contracted rather than skills being externally outsourced, and a strong enabler of HipHop2SToP was the employment of local community members to support the camp and music project. While this was a strengths-based approach, our findings support the need for appropriate mechanisms for direct employment rather than outsourcing via third-party contracts, as this did result in confusion around roles, expectations, and responsibilities (20). We confirm virtual connection was an essential component of HiPHop2StoP but due to the large number of stakeholders and people involved, gaps in communication were experienced. Participants described it being difficult at times for everyone involved to know what was going on or how tasks were progressing. Aligning with previous research, clear communication and clarity of roles and responsibilities (20) from the outset should be emphasised for future initiatives. Establishing formal agreements between stakeholders when projects commence may help to mitigate these challenges.

Our findings also revealed participants’ disappointment regarding post-production. Unlike previous Indigenous Hip Hop Projects where music videos are generally launched on the final day of production to celebrate the work, competing priorities of the media organisation producing HipHop2StoP along with COVID-19 travel restrictions, presented unfortunate circumstances post-production, leading to no official launch. Despite this, access to the video via YouTube has ensured frequent viewings by individuals, community groups and the wider public. The video has also been viewed in healthy skin educational workshops in schools across the region and shown at a range of cultural and scientific presentations about healthy skin.

### Limitations and strengths

While a small sample size of eight participants, this study provides insights from stakeholders and breadth of experiences of those involved in the music project and camp. Unfortunately, given the continuing COVID-19 community closures, there were no formal interviews conducted with the young people involved in HipHop2SToP, however several anecdotal reflections from adults have been included here. This paper contributes unique insights to the body of literature describing community led approaches to health promotion and presents novel ways of planning and implementing projects during the COVID-19 pandemic.

There was an overwhelming sense of gratitude from the stakeholders involved in the HipHop2SToP project; firstly, for it to be community led with involvement from approximately 15 local stakeholders and secondly, to observe an initiative that they believed ‘empowered’ the young children of the Dampier Peninsula. While HipHop2SToP defied the challenges of COVID-19 travel restrictions, lessons learnt reveal the ongoing need for clear and effective communication in health programs in addition to developing appropriate mechanisms for organisations to employ local community members. HipHop2SToP is an exemplar of Aboriginal leadership ensuring a strong sense of culture emanated throughout HipHop2SToP and this evaluation research project. While future health promotion music projects may not experience the challenges of COVID-19 restrictions, they would benefit from adopting this community led approach and negotiating roles and responsibilities clearly from the outset. Strengthening post-production outcomes by including a launch and well-planned, targeted communication and dissemination strategy will ensure the wider translation of important health messages and likely sustainability.

To view HipHop2SToP please click on the lick at: https://www.youtube.com/watch?v=7eLLO9EuOiI.

## Data availability statement

The raw data supporting the conclusions of this article will be made available by the authors, without undue reservation.

## Ethics statement

The studies involving humans were approved by Child and Adolescent Health Service (Approval number RGS0000000584), the Western Australian Aboriginal Health Ethics Committee (Reference number: 819), University of Western Australia (Reference RA/4/20/4123), Catholic Education Western Australia (Reference number: RP2017/57) and Western Australian Department of Education (Reference number: D18/0281633). The studies were conducted in accordance with the local legislation and institutional requirements. The participants provided their written informed consent to participate in this study. Written informed consent was obtained from the individual(s) for the publication of any potentially identifiable images or data included in this article.

## Author contributions

TM: Conceptualization, Data curation, Formal analysis, Investigation, Methodology, Writing – original draft. RW: Conceptualization, Supervision, Writing – review & editing. SE: Formal analysis, Writing - review & editing. HT: Writing – review & editing. JJ: Project administration. SS: Project administration. MM: Project administration, Writing - review & editing. NM: Project administration. JCo: Supervision, Writing – review & editing. JCa: Supervision, Writing – review & editing. AB: Funding acquisition, Supervision, Validation, Writing - review & editing.

## Footnotes

^1^At the time of HipHop2SToP, Community Navigators are community members working alongside family groups and leading community consultation, stakeholder engagement and co-design processes to enhance community mobilization around key social issues as well as facilitate culturally competent, integrated, and holistic service delivery.

^2^Yarning is a culturally appropriate methodology for gathering data/information through story sharing and engaging with Aboriginal people.

^3^Kulunga Aboriginal Units’ role is to be the primary professional support service that links Aboriginal communities with researchers working at Telethon Kids Institute. Kulunga has a team based in Broome who oversee research conducted in the remote Kimberley communities.
